# National Culture and Culinary Exploration: Japan Evidence of Heterogenous Moderating Roles of Social Facilitation

**DOI:** 10.3389/fpsyg.2021.784005

**Published:** 2021-11-25

**Authors:** Bin Liu, Yang Wang, Sotaro Katsumata, Yulei Li, Wei Gao, Xi Li

**Affiliations:** ^1^Economics and Management School, Jiangxi Science and Technology Normal University, Nanchang, China; ^2^Greater Bay Area International Institute for Innovation, Shenzhen University, Shenzhen, China; ^3^Graduate School of Economics, Osaka University, Toyonaka, Japan; ^4^Business School, Durham University, Durham, United Kingdom; ^5^Japanese Department, School of Foreign Languages, Shenzhen University, Shenzhen, China; ^6^College of Management, Shenzhen University, Shenzhen, China

**Keywords:** travel companions, user-generated content, culinary consumption, uncertainty avoidance, individualism-collectivism

## Abstract

This article explores the role of national culture in the culinary consumption behavior of international tourists and the moderating influence of different types of travel companions. Study 1 adopted a text-mining, topical modeling approach to process useful reviews (*n* = 7,803) posted at TripAdvisor by users from 86 countries. Study 2 develops and tests a conceptual model of the relationships between national culture and culinary choices including culinary types and range of culinary choices, based on a secondary dataset of large-scale surveys from the tourism authority of the destination containing 9,141 responses by tourists from over 60 countries or regions. The results reveal that both Uncertainty Avoidance and Individualism-Collectivism have significant effects on tourist food consumption categories and the range of culinary choices. The study also evaluated the role of the moderating effect of travel companions, and results supported the significant relationship on the range of culinary choices when the tourists were accompanied by different types of travel companions.

## Introduction

Food is considered an essential part of a culture and the food preferences of tourists are shaped by their cultural background (Jia, 2020; Kim and Huang, 2021). Tourism scholars have generated valuable insights into national culture and tourist experiences (Seo et al., 2012; Li and Katsumata, 2020), by drawing on the framework of cultural dimensions by Hofstede (1980). However, most studies are limited to comparing tourists from two or three cultures based on relatively small samples and those based on the framework of Hofstede often focus on a single dimension of the framework (Jia, 2020). Moreover, despite the interesting findings reported in the extant literature, little is known about how the variations along the cultural dimensions influence tourist exploration of local food at a foreign destination.

The Japanese culture emphasizes experiencing a variety of authentic cuisines closely related to a destination, thus, Japan promotes its tourism industry by marketing specialty seasonal food products known as Meibutsu. Regarding Japanese cuisines, according to Japanese tourism authorities and previous studies (Kim, 2015; Kim and Ellis, 2015; Japan National Tourism Organization, 2020; Osaka Convention and Tourism Bureau, 2020), Japanese daily cuisines including Japanese noodle, Takoyaki, Okonomiyaki, Kushikatsu, Yakitori, Izakaya food, and Bento as Japanese casual daily cuisines, while Sukiyaki, Teppanyaki, Kaiseki, and Kappo are segmented into traditional Japanese luxury cuisines. According to statistics from Osaka Convention and Tourism Bureau, the consumption rates of Japanese casual cuisines by tourists were Ramen (67%), Takoyaki (51%); while Japanese luxury cuisines were less popular, including Sukiyaki (9%), Shabushabu (8%), Kaiseki (7%), and Kappo (3%) (see Table 1).

**TABLE 1:** Cuisines and consumption rates in Osaka, Japan.

Categories	Description	*n*	%	Japanese luxury cuisine	Japanese daily cuisine	Total cuisines
Ramen	Japanese noodle	3062	67		✓	✓
Sushi	It is made of raw fish, shrimp, shell, etc., combined with rice, and pressed by hand	2462	54	✓		✓
Takoyaki	Osaka fast food and sold at roadside stalls	2329	51		✓	✓
Udon, Soba	Japanese noodle. Udon is made of thick wheat flour; Soba is made of buckwheat	1830	40		✓	✓
Tempura	Battered and deep-fried vegetables and seafood	1476	33		✓	✓
Okonomiyaki	Japanese savory pancake or Japanese pizza	1298	29		✓	✓
Yakiniku	Luxury beef, including Kobe beef and Matsuzaka beef	1278	28	✓		✓
Seafood	Japanese style seafood including Fugu (blowfish), crab, sashimi	1084	24	✓		✓
Bento	It is a common ingle-portion take-out meal	1004	22		✓	✓
Yakitori	Skewered chicken, sometimes offering chicken sashimi	810	18		✓	✓
Izakaya food	Informal Japanese bar that serves simple food and alcoholic drinks	601	14		✓	✓
Kushikatsu	Deep-fried panko crusted meat and vegetable	577	13		✓	✓
Sukiyaki	Luxury Japanese hotpot, usually served with rare egg and sweet sauce	431	9	✓		✓
Shabushabu	Japanese hotpot served with dipping sauces, which is more savorer and less sweet compared with Sukiyaki	364	8	✓		✓
Kaiseki	The sophisticated way of Japanese dining, usually elaborately prepared cuisines served artistically in a formal dining room	305	7	✓		✓
Kappo	Multi-coursed high-end Japanese cuisine	122	3	✓		✓

*Respondents reported the categories among the above 16 listed restaurants (1 = yes, 0 = no) of dining during their stay in Osaka. The source of description: Osaka Convention and Tourism Bureau and Japan National Tourism Organization.*

The cultural framework created by Hofstede has been widely applied in various studies of international business and management to explain a range of phenomena, including the difference between countries/regions at the national, organizational, and individual levels. From the micro perspective, most studies focused exclusively on Uncertainty Avoidance (UAI) and Individualism-Collectivism (IDV), two dimensions that have become widely accepted in different fields of studies and have generated stable and reliable results at individual outcomes (Shavitt and Barnes, 2020; Watts et al., 2020). In food tourism literature, Jia (2020) found a link between individualism/collectivism and restaurant evaluation as U.S. tourists (individualism) tend to focus on occasion-related topics, while Chinese tourists (collectivism) tend to discuss more food-related topics. From the macro perspective, a series of reviews of the literature suggests that “Power Distance,” and “Masculinity-Femininity” have theoretical relevance for the empirical research at the organization or/and country levels (Kirkman et al., 2006, p. 310, 2017), and as this study focuses on the individual level, these two dimensions are not examined in this study. Additionally, “Indulgence versus Restraint” is theoretically relevant at the organization level as well and relatively less applied to empirical studies, and “Long-term versus Short-term Orientation” is most relevant to Asian cultures (Dimitratos et al., 2011), not suited for studies focusing on multinational tourists. Therefore, these two dimensions are not examined in this study, either.

**FIGURE 1:**
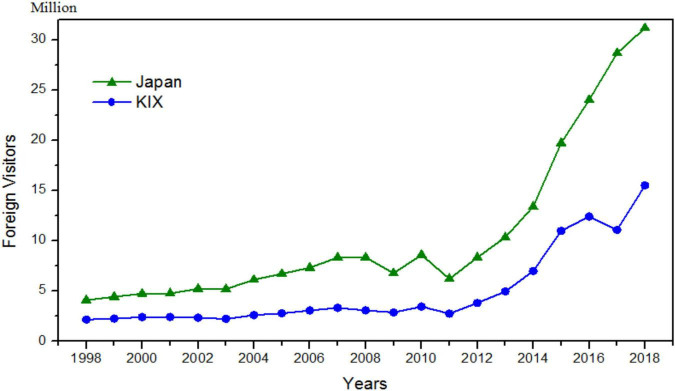
Trends of foreign visitors to Japan and Kansai International Airport (KIX). Osaka Convention and Tourism Bureau (OCTB), and New Kansai Airport Company Co., Ltd.

Unlike many cultures and tourist behavior studies that examine only a single culture dimension and rely on rather small samples of tourists from two or three cultures, we simultaneously examine two dimensions (UAI and IDV). In Study 2, the research context of this study is Osaka (Japan), we employ a large sample (*n* = 9,141) of data collected over 2 years by the Osaka Convention and Tourism Bureau (OCTB), and the results can closely reflect the diversity of international tourist. The findings of this study have practical implications for destination marketing organizations to promote local food (Rousta and Jamshidi, 2020), and in turn, food image provides tourists with extradentary and memorable local culinary experience (Kim and Huang, 2021). Osaka is situated near the sea and has been a commercial center since the Edo period, together with fish and seashells are abundantly available, and cuisines are easily transportable from other areas, Osaka has been named the “Kitchen of the Nation” in Japan. More recently, Osaka city as the capital of Osaka Prefecture ranked as the third and fourth most livable of the 140 cities surveyed by The Economist Intelligence Unit in 2018 and 2019, respectively (The Global Liveability Index, 2019). It is the host city for the 2025 World Expo. Osaka is a critical transportation hub linking Kyoto and Nara, the cultural capital of Japan, and attracts tourists from over the world. The number of international tourists traveling through the Kansai International Airport in Osaka is roughly half of all international tourists to Japan in the past two decades (see Figure 1).

The cultural framework created by Hofstede is considered the classic theory for analyzing cultural differences and tourist behaviors (Li and Katsumata, 2020; Li J. et al., 2021). The cultural dimensions have been applied for developing insights into the relationships between culture and tourist experiences and satisfaction. Jia (2020) who find a link between individualism/collectivism and restaurant evaluation, i.e., U.S. tourists (individualism) tend to focus on occasion-related topics, while Chinese tourists (collectivism) tend to discuss more food-related topics. However, little is known about the impact of cultural dimensions on the culinary consumption of international tourists, such as the differences in food-related topics in social media, consumption of category-specific cuisines (e.g., luxury versus daily), and the range of culinary choices. Moreover, the role of activity companions has been well studied in retailing, including enhancing the perceived hedonic value and relieving the anxiety of consumers (Li X. et al., 2021) and stimulating consumption (Clauzel et al., 2019); and influencing the emotional arousal of tourists (Su et al., 2020). However, the role of different types of travel companions in tourism literature is limited, while such knowledge is important not only for tourism organizations but also the core element of considering re-design of service in hospitality.

The main objective of this study was to bridge the aforementioned gap in tourism literature by empirically examining the impacts of national culture on culinary experiences. Specifically, we examined the following. Firstly, we examined whether national culture impacts tourist category-specific culinary consumption (luxury vs. daily) and the range of food choices. Secondly, if so, we investigated how these effects are moderated by the different types of travel companions. Two empirical studies were designed. Study 1 aimed to explore the similarities and differences of culinary experiences by the two cultural dimensions of UAI and IDV. In Study 2, we proposed several hypotheses related to the relationships among national culture, culinary consumption, and travel companions.

This research contributed to the tourism literature in several ways. First, we provided new evidence of cross-cultural differences and the consumption experiences of tourists of local food at the destination, as well as revealed the effect of travel companions on the relationship between cross-cultural differences and the consumption experiences of tourists of local food at the destination. Even tourists from high-UAI cultures, under the context of being companied by friends, are apt to consume a greater range of food and daily cuisines. Moreover, this research extended the literature by showing tourists from collectivistic cultures traveling with family members, friends, and colleagues showed a greater range of food choices than those traveling alone, and which further leads to enhance satisfaction and revisit attention. Second, we developed a conceptual framework between national/regional culture and tourist consumption of local cuisines in the specific aspects of choice of luxury cuisines versus daily cuisines and the range of food choices. Tourists from high-UAI cultures are more likely to consume luxury cuisines, whereas driven by curiosity, those from lower ones are more likely to be novelty-seeking in food by exploring the local cuisines and inexpensive daily food in the local markets (Hsieh and Chang, 2006). Third, we offered a methodological contribution by synthesizing two empirical studies using different data sources: TripAdvisor and the official tourist survey of the destination tourism authority in addressing the research problem. In design is innovative and robust in that it adopts a machine learning netography approach and triangulates the findings with those from a secondary dataset of large-scale surveys from the tourism authority of the destination. We provided a new, detailed framework on how to use hierarchical Latent Dirichlet Allocation and several other algorithms supplemented with manual interpretation to derive insightful findings from User-Generated Content. Finally, the results of this study offered important implications for destination marketing.

## Literature Review

### Cross-Cultural Factors and Food Tourism

Food is considered an essential part of a culture, and the food preferences or evaluations of tourists are shaped by their cultural background (e.g., Jia, 2020). Food consumption is not just to satisfy our physiological needs but also to social and psychological needs. Dining provides opportunities for individuals to socialize, and to develop a sense of cultural identification, which is also a source for developing cultural links between different countries (Jia, 2020).

The study of Hofstede (1980) initially proposes four cultural dimensions in the cultural framework: “Power Distance,” “Uncertainty Avoidance,” “Individualism-Collectivism,” and “Masculinity-Femininity.” The work of Hofstede (2001) later adds the Confucian dynamic of “Long-term versus Short-term Orientation” and combines the dimension of “Indulgence versus Restraint.” As argued by Hofstede (2001), these cultural dimensions are the shared assumptions among members of a particular cultural group, and people from different cultural backgrounds will have different views about what is appropriate in various situations. Our work focuses on the two dimensions of Uncertainty Avoidance and Individualism-Collectivism.

Uncertainty Avoidance refers to the extent to which uncertainty is undesirable and people attempt to avoid it (Hofstede, 2001). Members of high-UAI cultures feel threatened with uncertain or unknown environments and have a strong need for clarity and security. Formality, written rules, or unwritten social norms provide certainty and a sense of security (De Mooij and Hofstede, 2002). Compared with people from low-UAI cultures, they are less open to new experiences and appear to be conservative. In the context of gastronomic consumption, high-UAI consumers are less likely to try new cuisines, have a limited range of food choices (Tse and Crotts, 2005). However, the research of Seo et al. (2012) find that foreign expatriates in South Korea who are from high-UAI cultures tend to perceive Korean foods as expensive and dine out less often than those from low ones, and they do not find significant differences between the two groups in attitudes, understanding, preference, satisfaction, and behavioral intentions with regards to Korean food or restaurants.

Individualism-Collectivism refers to the degree to which the welfare of the individual is valued more than the group (Hofstede, 2001). Members of the individualistic society are “me-oriented”). Their personal interests are seen as more important than group interests. They see themselves as independent from each other. The social networks in individualistic societies are rather loose. Members value their personal belief and their behaviors are guided more by their own interests and preferences than group ones (Oyserman et al., 2002). In contrast, members of collectivist society are “we-oriented,” they value the interests and social norms of their group. The social networks are fairly tight in a collective society, collectivists value the opportunity of interpersonal interaction and group membership is an important aspect of their identity (Gilboa et al., 2020). In addition to themselves and immediate families, collectivists also care about friends and other members of their group. Their behaviors are usually guided by group expectations and social norms (Torelli and Shavitt, 2010; Cao et al., 2021; Zhou et al., 2021). Collectivistic cultures emphasize the positive emotional value of culinary consumption on social occasions as a driving force of negotiating and fostering social relationships, bridging communication, and reinforcing a sense of belonging (Kim et al., 2020). Specifically, dining out together when traveling abroad can deepen interpersonal affection (Kim and Eves, 2012), and to the benefit of building new social relations and strengthening social bonds (Mak et al., 2012).

Overall, several studies have compared Western tourists and Asian tourists and suggested that Asians are less willing to challenge novel and unfamiliar cuisines while traveling abroad. The study of Cohen and Avieli (2004) indicate that Asians tend to be less novelty-seeking and more dependent on restaurants that provide their home cuisines. Japanese tourists are less inclined to culinary novelty-seeking when compared with American and Canadian tourists (Sheldon and Fox, 1988), and Koreans have a strong preference for their national cuisines (March, 1997). Within the western cultures, French customers (high-UAI) are more accepting of recommendations from waiters than those from English-speaking countries who (low-UAI) prefer to make consumption decisions by themselves (Cohen et al., 2009). Nevertheless, there are variations among Asian or Western cultures and food consumption behavior.

The cultural framework created by Hofstede has been widely applied in various studies of international business and management to explain a range of phenomena, including the difference between countries/regions at national, organizational, and individual levels. From the micro perspective, most studies focused exclusively on UAI and IDV, two dimensions that have become widely accepted in different fields of studies and have generated stable and reliable results at individual outcomes (Sekiguchi, 2006; Shavitt and Barnes, 2020; Watts et al., 2020). In food tourism literature, Jia (2020) found a link between individualism/collectivism and restaurant evaluation as U.S. tourists (individualism) tend to focus on occasion-related topics, while Chinese tourists (collectivism) tend to discuss more food-related topics. From the macro perspective, a series of reviews of the literature suggests that “Power Distance” and “Masculinity-Femininity” have theoretical relevance for the empirical research at the organization or/and country levels (Kirkman et al., 2006, p. 310, 2017), and as this study focuses on the individual level, these two dimensions are not examined in this study. Additionally, “Indulgence versus Restraint” is theoretically relevant at the organization level as well and relatively less applied to empirical studies, and “Long-term versus Short-term Orientation” is most relevant to Asian cultures (Dimitratos et al., 2011), not suited for studies focusing on multinational tourists. Therefore, these two dimensions are not examined in this study, either.

### Travel Companions and Their Impact on Culinary Experience

The presence of a travel companion can impact tourist behavior and decision-making in a foreign unfamiliar destination, travel companion can assist in decision-making through providing information and mental support, which reduces risk perception and enhance satisfaction. The travel companion might be friends or peers, family members, and colleagues, etc. Significantly research had examined the differences in behaviors when the consumer is alone versus with someone. In the Marketing literature, the impact of shopping companions has been well examined, including the influence of spending (Yim et al., 2014), shopping experience (Li X. et al., 2021). Additionally, the study of Borges et al. (2010) identified the type of shopping companion significantly impacts on hedonic shopping value, shoppers express more positive emotional effect and hedonic shopping motivation of accompanied by friends in relation to either shopping with family members or alone. In the context of tourism, travel companions have been repeatedly examined as a control variable (Li and Katsumata, 2020). Little research qualifies the mediating or moderating effects of travel companions (Su et al., 2020). More specifically, the outcome of differences among the type of travel companions has been neglected in the tourism literature. Thus, this research questions the moderating effects of travel companions on the relationship between national culture and culinary consumption by qualifying the type of person tourist travels with, e.g., partner vs. family vs. friend vs. colleague.

## Study 1: Exploring Tourist Culinary Experience Through Social Media Analytics

Study 1 attempted to provide a better understanding of what types of culinary experiences matter most to tourists from different cultural backgrounds. Specifically, we applied the User-Generated Content posted on the travel platform TripAdvisor.com to answer the following two research questions.

(a) *Are there any differences in culinary experiences between tourists from high and low Uncertainty Avoidance groups?*
(b) *Are there any differences in culinary experiences between tourists from high and low Individualism-Collectivism groups?*

### Social Media Analytics in Tourism Research

The breathtaking growth of social media has attracted increasing attention from researchers in marketing and tourism (Jia, 2020; Sun, 2021; Sun et al., 2021). Tourism scholars focus on three fields regarding the content: “What is the mining”; “who post the content”; and “why do they post?” (Leung et al., 2019). Due to the nature of our study, we explore the “who posts the reviews” (what cultures those tourists are from) and “what are the reviews” (various experiences of tourists). Previous studies have proposed multiple frameworks for these two tasks. For example, the study of Vu et al. (2019) utilize restaurant reviews to analyze tourist dining preferences. The recent development of machine learning and artificial intelligence models enables researchers to deal with the limitations of traditional methods. Various Natural language processing models are available as well, such as sentiment analysis (Alaei et al., 2019) and topic modeling (Jia, 2020). However, the results generated from machine learning models are at an aggregated level, losing the fine details at the individual, micro-level, which are valuable to generate in-depth knowledge. Moreover, although the sample sizes of social media contents are normally much larger than those collected from traditional research methods, yet the representativeness of the samples is limited to those who posted the comments, while those who did not are missed out. Therefore, our research integrates both traditional and machine learning methods to complement each other.

### Data Crawling and Cleaning

A total of 36,091 pieces of User-Generated Content commenting on restaurants in Osaka were retrieved by applying ScrapeStorm (2019), a crawler software. Each review contained *restaurant names, cuisines, review comments, user location*, whereas topics are not relevant to the restaurant and culinary services were removed, and reviewers whose places of origin are Japan are further removed. After the data cleaning, 7,803 reviews remained which were posted by tourists from 86 countries. The data was divided according to the places of origin of the reviewers into four cultural groups using the two dimensions of UAI and IDV. More specifically, the median of the cultural score calculated from 86 countries in this study, if the assigned score of a country is higher than the median of the assigned culture score, we categorize the country as high-UAI (IDV) group. Table 2 shows the locations and proportion of reviewers, and Table 3 shows the data distribution of these four groups.

**TABLE 2:** The top 20 locations and proportions of reviews.

Country	No. of reviews
United States	1272
Singapore	1007
Australia	895
Great Britain	605
Hong Kong	584
Philippines	382
Malaysia	354
Canada	335
Thailand	291
Indonesia	231
China	177
Korea South	145
Italy	136
Switzerland	129
Taiwan	129
France	118
Netherlands	109
Spain	106
Germany	97

**TABLE 3:** Data distribution by cultural groups.

Cultural groups	No. of reviews	Proportion (%)
High-UAI	812	10
Low-UAI	7161	90
High-IDV	4208	53
Low-IDV	3765	47

### Data Analysis

The unstructured review data were transformed to generate the *corpus* and *vocab* by performing a series of Natural Language Processing procedures as follows. First, we only extracted reviews posted in English as the hierarchical Latent Dirichlet Allocation algorithm could only process English. This step was followed by using the text tokenization algorithm in the Natural Language Processing package (Loper and Bird, 2002), in which the reviews were split into words called tokens. An algorithm was applied to convert all tokens to lower case and the PortStemmer module in the package converted those tokens into root form. For example, “service,” “servicing,” “services,” and “serviced” mean the same thing, accordingly, stemming those words into “service” can improve the performance of the topic modeling algorithm. According to Vu et al. (2019), English noun words normally refer to interested entities, and therefore only nouns have been retrieved from the stemmed tokens. Further, the stop words were removed, which are normally in English sentences, whereas most of the time are meaningless for User-Generated Content analysis, such as “will,” “you,” “I,” etc. The Natural Language Toolkit package (Loper and Bird, 2002) collected usual stop words and we added some extra words into the list, such as the name of the restaurant, “place” and “restaurant.” After that, the tokens were loaded to the *corpus* and *vocab* variables and utilized in the topic modeling.

In this study, hierarchical Latent Dirichlet Allocation was adopted to explore User-Generated Content commenting on restaurants in Osaka by the international tourists who reside in different outbound markets. Hierarchical Latent Dirichlet Allocation is a non-parametric extension of Latent Dirichlet Allocation developed by Blei et al. (2010). It is an unsupervised topic modeling algorithm to cluster natural languages into various topics, such as reviews or comments. Hierarchical Latent Dirichlet Allocation has been commonly used in the marketing disciplinary (Toubia et al., 2019; Jia, 2020) and with two advantages compared with the traditional one: (a) instead of manually providing the topic number *K* in Latent Dirichlet Allocation algorithm, hierarchical Latent Dirichlet Allocation can generate topics automatically without given any topic number parameter; and (b) hierarchical Latent Dirichlet Allocation algorithm can present the topics in a hierarchical structure. In this study, we used the hierarchical Latent Dirichlet Allocation package written by Reades (2019) in Python 3. The final results were then transformed and visualized in the work of Flourish (2020).

### Findings

**FIGURE 2:**
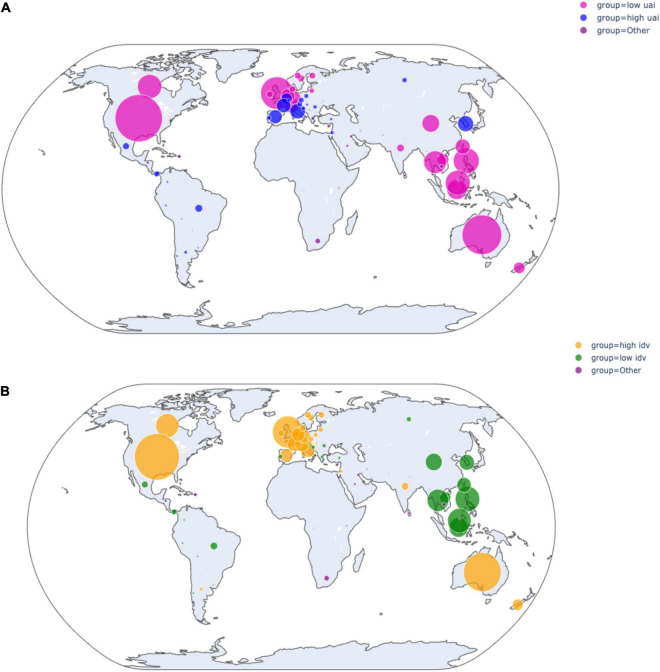
**(A)** Distributions of reviews from high and low-UAI. **(B)** Distributions of reviews from high and low-IDV.

By adopting scored cultural dimensions of Hofstede (2015) based on the home cultures of reviewers, we utilized the algorithm to classify the reviews in four different cultural groups: high/low-UAI; high/low-IDV. The distribution of the User-Generated Content is shown in Figures 2A,B.

#### Culinary Experience of High/Low Uncertainty Avoidance Group

##### High Uncertainty Avoidance Group

**FIGURE 3:**
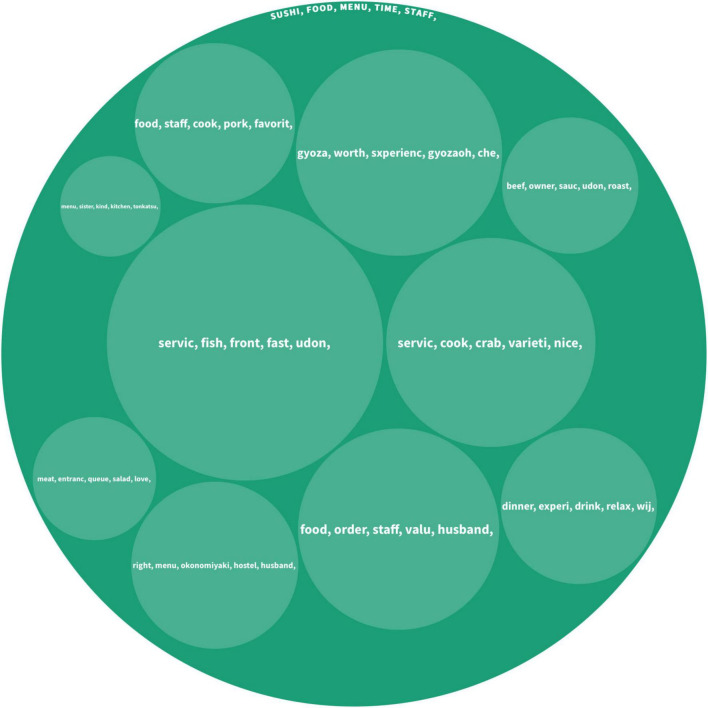
Topics discussed by the group high-UAI.

This group contained 812 pieces of User-Generated Content from 32 countries/regions, and the detailed information is shown in Figure 3. The top five keywords under the main topics are including *sushi, food, menu, time*, and *staff*. The keywords indicate that *sushi* is the most popular cuisine among tourists from high-UAI cultures. The *menu* is another important keyword when foreign tourists reviewed those restaurants. Most tourists mentioned whether they can understand the Japanese language and choices on a menu. The keyword *time* mainly refers to the frequency of enjoying the favorite food of an individual. *Staff* is mainly indicating service quality.

The sub-topics extracted from the hierarchical Latent Dirichlet Allocation algorithm show that other major topics discussed by the high-UAI group include *fish*, *beef*, *crab*, *Udon*, and *Gyoza* (dumplings). Tourists also frequently mentioned their hedonic culinary experience including words such as *wonderful*, *nice*, *relaxed*, *love*, *favorite*, and *right*. For example, an Italian tourist posted, “The sushi was wonderful and handmade in front of our eyes!”. Moreover, tourists in this group frequently mentioned travel companions within sub-topics. A Belgian tourist reviewed as “Maybe it’s just our taste, but neither my husband and I liked it.”

##### Low Uncertainty Avoidance Group

**FIGURE 4:**
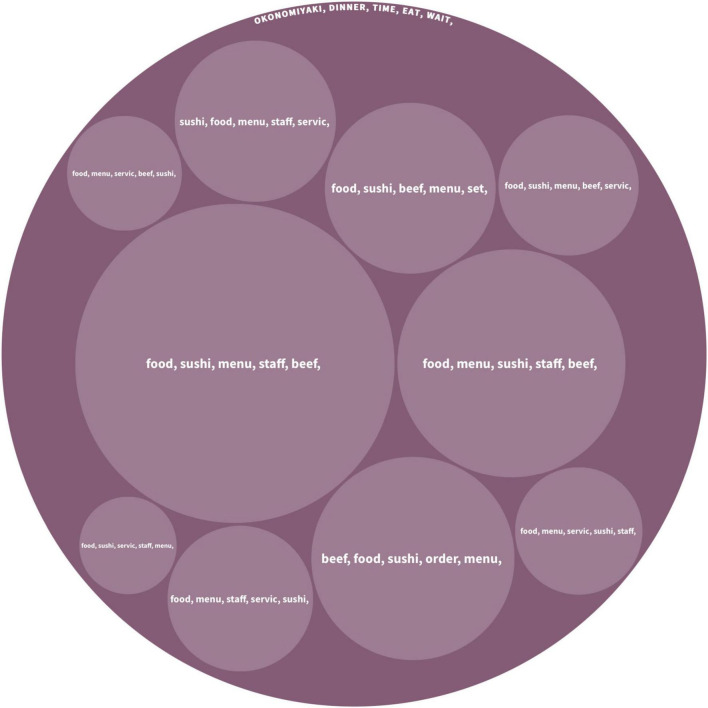
Topics discussed by the group of low-UAI.

This group contained 812 pieces of comments from 54 countries/regions, and Figure 4 visualizes the hierarchical structure of the topics generated from the hierarchical Latent Dirichlet Allocation algorithm. The top five keywords under the main topics are including *okonomiyaki*, *dinner*, *time*, *eat*, and *wait*. It shows that tourists from low-UAI cultures are more likely to consume the okonomiyaki (a popular Japanese street food). The meaning of the three keywords *dinner, time* and *eat* are straightforward and similar to the keywords discussed by high-UAI tourists. Moreover, tourists in this group frequently mentioned *waiting*, which is mainly about the waiting time of being served including the queuing time. According to the results of the hierarchical Latent Dirichlet Allocation algorithm, this group also contains sub-topics, including *food*, *sushi*, *menu*, *staff*, and *beef*.

##### Comparison Between High/Low Uncertainty Avoidance Groups

The two groups showed different food preferences. According to the classification in Table 1, the high-UAI group focused on luxury cuisines such as fish, beef, and crab, whereas the low ones focused on daily local cuisines, such as *okonomiyaki*. Moreover, the high-UAI group seemed to dine out with travel companions (e.g., sister or husband) and had a greater range of food discussed than the low ones, such as *fish*, *udon*, *crab*, *pork*, and *gyoza*. Nevertheless, there are several common topics in both groups, such as staff and customer services.

#### Culinary Experience by High/Low Individualism-Collectivism

##### Individualism Group

**FIGURE 5:**
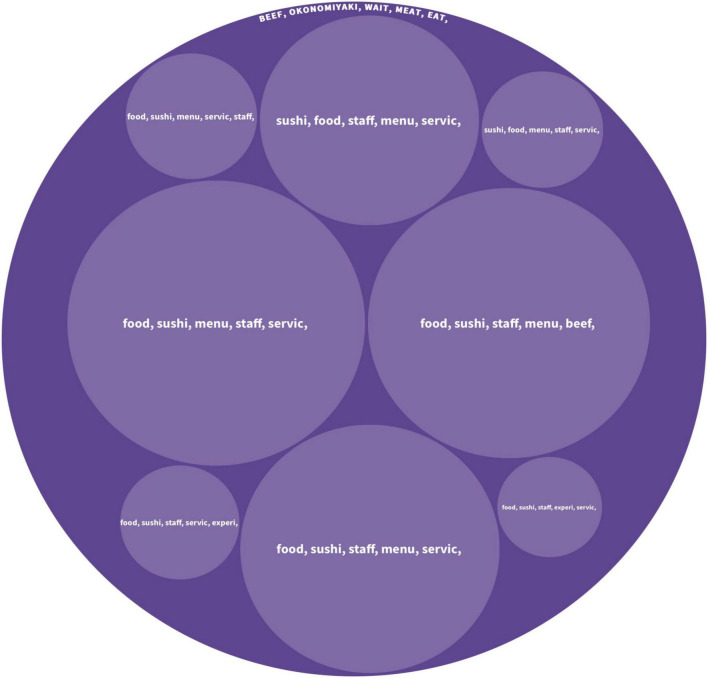
Topics discussed by the group of high-IDV.

This group contained 4,298 pieces of User-Generated Content from 30 countries/regions, and Figure 5 presents the topic visualization. The keywords in the root topics discussed by this group are *beef*, *okonomiyaki*, *wait*, *meat*, and *eat*. The most popular Japanese cuisines for this group are *beef* and *okonomiyaki*. According to the User-Generated Content, the two most popular and expensive kinds of beef are Matsuzaka Beef and Kobe Beef. *Okonomiyaki* is one of the most popular cuisines among tourists in collectivist groups. The results also showed that, for the individualism group, waiting time is the major issue in their culinary experience. In addition to the main topic, the sub-topics show that the tourists in this group paid much attention to staff and services.

##### Collectivism Group

**FIGURE 6:**
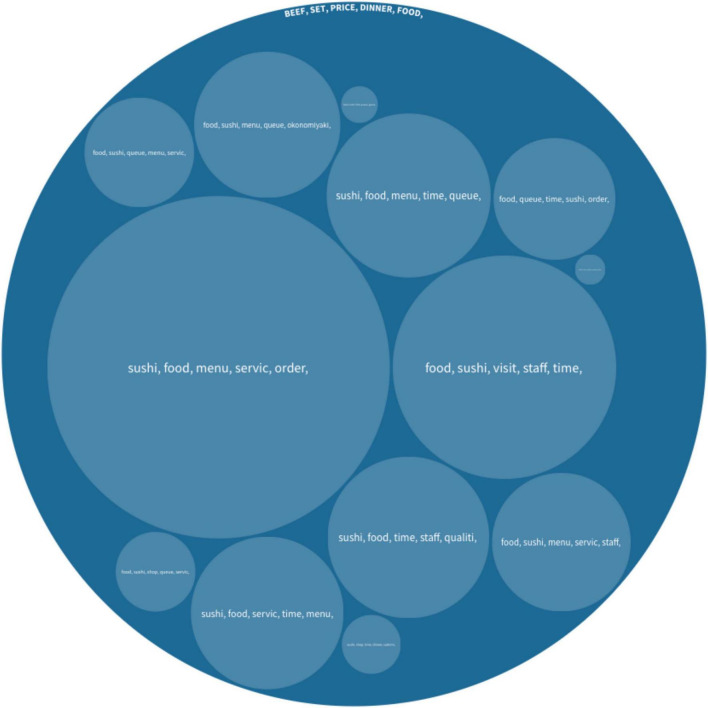
Topics discussed by the group of low-IDV.

This group contained 3,765 pieces of User-Generated Content from 56 countries/regions. Keywords under the main topics are *beef*, *set*, *price*, *dining*, and *food*. Figure 6 presents the topic visualization. The results indicate the most popular food is *beef*. The second keyword *set* suggests that this cultural group focuses on the food in the set. The keyword *price* suggests that price is another hotly discussed topic. The sub-topics suggest that tourists from collectivistic cultures include *menu*, *customer service*, and *placing orders*, which are largely related to language and customer services.

##### Comparison Between High/Low Individualism-Collectivism Group

The differences between these two cultural groups are noteworthy. The root topics of the two groups indicate that tourists from individualistic cultures pay much attention to the quality of the food and the waiting time before they were served. However, collectivism focuses more on economic aspects, such as price or set, than the other group does.

Study 1 revealed the various topics discussed by tourists from different cultures. However, the data are limited to User-Generated Content in English, leaving out those in other languages. In addition, the level of statistical significance for the differences is not known. Study 2 aimed to develop and empirically test the relationships between the two cultural dimensions and the culinary consumption behavior of international tourists.

## Study 2: Hypothesis Development and Empirical Testing

**FIGURE 7:**
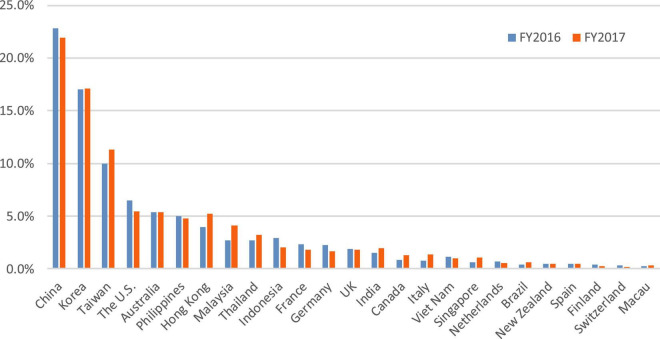
Share of nations/regions of international tourists to Osaka. Total samples of FY2016 (*n* = 4,474) and FY2017 (*n* = 4,647); FY, fiscal year.

Study 2 developed and tested a conceptual model of the culinary experiences of international tourists by the two cultural dimensions of UAI and IDV with different types of companions (partner versus family versus couple versus friend versus colleague), as shown in Figure 7. We focused on the category-specific culinary consumption of international tourists (luxury versus daily cuisines) and the range of culinary selection.

### Hypothesis Development

#### Cultural Influence and Culinary Consumption

As discussed earlier, previous studies have suggested that tourists from different cultural backgrounds show different food preferences at a travel destination. Since tourists from risk-avoiding cultures have a strong need for clarity and security in an unfamiliar environment, traditional restaurants that offer formal dining, purity in food, or luxury cuisines are more favored than the informal dinner environments such as street food and night markets (De Mooij and Hofstede, 2002). They may try to reduce risk by consuming familiar cuisines, tourism-oriented food, or luxury cuisines in a sheltered environment (Cohen and Avieli, 2004). Since tourists from novelty-seeking cultures tend to show high interest in local dishes, visiting local markets and eating at roadside stalls or night markets, which indicates that they are likely to consume daily cuisines at overseas travel destinations.

H1a: *Tourists from high*-*UAI cultures are more likely to consume luxury cuisines.*

Tourists from low-UAI cultures are more likely to “play with risk,” and more likely to be attracted by local cuisines at venues such as night markets, which are mostly inexpensive daily food (Hsieh and Chang, 2006). Pizam and Sussmann (1995) find that tourists from Japan, France, and Italy tend to avoid local food at travel destinations, while those from the U.S. tend to patronize local restaurants. Such results seem to be correlated with the ratings of UAI: Japanese, French, and Italian have a high-UAI score than Americans (Hofstede, 2015). According to the cultural framework, tourists from low-UAI cultures are more risk-seeking, enjoy adventure-seeking activities, thus they are likely to try various cuisines (Tse and Crotts, 2005), thereby increasing their culinary variety-seeking behaviors at overseas travel destinations. Therefore, we hypothesized that:

H1b: *Tourists from low-UAI are more likely to consume daily cuisines.*
H1c: *Tourists from low-UAI are more likely to increase their range of food consumption.*

The motivation of tourists behind dining out is highly influenced by their lifestyles and cultural backgrounds (Chang et al., 2011). Members of collectivist societies are recognized as “we-oriented,” and they value more the interests and social norms of their group than personal ones. Collectivists also value the opportunity of interpersonal interaction and group membership as an important aspect of their identity (Bakir et al., 2020). Apart from their immediate families, collectivists also care about friends and other members of their group, and their behaviors are usually guided by group expectations and social norms (Torelli and Shavitt, 2010). Thus, tourists from collectivistic cultures may take the culinary preferences of their travel companions into their own consumption sets and increase the culinary consideration sets, and thus leading to greater culinary variety-seeking behaviors. Accordingly, we proposed the following hypothesis:

H2: *Tourists from collectivistic cultures are more likely to increase their range of culinary selections.*

#### The Moderating Effect of Travel Companions

Social impact theory indicates that other people present in each context will impact the decision-making of individuals (Latané, 1981). Most dining takes place in the presence of companions, and therefore social factors significantly affect dining, such as dining companions (Cruwys et al., 2015). In both the travel and the daily settings, food consumption is not only to satisfy our physiological needs, which is also inherently a vital social interaction experience, and social influence has long been recognized as a key factor of impacting the consumption decisions of individuals (Yim et al., 2014). Specifically, in the travel set, the main role of dining is for social and psychological needs. For example, dining provides opportunities for individuals to socialize, and to develop a sense of cultural identification in the travel destinations (Jia, 2020). Travel activities are accompanied by a greater level of uncertainty than daily lives, but the presence of a travel companion alleviates the anxiety of accompanied tourists, and in turn positively influences participation in challenging tourism activities (Su et al., 2020). Specifically, for tourists, dining at unfamiliar travel destinations may be full of uncertainty. In particular, tourists from high-UAI cultures are less risk-taking, avoiding adventure-seeking activities, and thus they are less likely to try unfamiliar or high-risk cuisines, such as night market food, when traveling overseas (Tse and Crotts, 2005). The presence of companions can prompt impulse consumption (Yim et al., 2014), and thus travel companions may prompt tourists to try unfamiliar or risky cuisines. Moreover, previous studies have identified that higher exposure to social stimulus in the marketing environment leads to an increase in the likelihood of impulse consumption (Clauzel et al., 2019; Chen et al., 2021; Katakam et al., 2021). Bevelander et al. (2011) uncover that the peers induced willingness to try unfamiliar cuisines. Thus, we proposed the following hypotheses:

H3: *UAI has a stronger effect on daily cuisines consumption in the presence (vs. absence) of travel companions.*
H4: *UAI has a stronger effect on the range of culinary consumption in the presence (vs. absence) of travel companions.*

Previous research identified that the larger the group of diners, the higher the individual spends (Clauzel et al., 2019), and people tend to present a positive social image in the presence of others (Otterbring, 2021). Tourists from collectivistic cultures tend to place greater emphasis on companions. For example, China is classified as a collectivistic culture and Chinese tourists tend to accomplish activities in a group and thus show greater tolerance to crowdedness than tourists from individualistic cultures (Kim et al., 2010). In a collectivist culture, dining out with family members, friends, and colleagues is an important means for building and maintaining social relationships. Those from a collectivist culture pay more attention to building a close-knit framework, harmony, and emotional attachment by getting along with the group and avoiding being alienated (Hui and Triandis, 1986). Compared with solo travelers, group travelers need to make collaborative consumption decisions and based on the social facilitation theory, group tourists tend to balance individual preferences in the group consumption decision-making process (Su et al., 2020). The study of Herman et al. (2003) found that groups can encourage individual culinary consumption. Additionally, under the case of individuals more concerned with maintaining social harmony, and in turn, the influence of social factors increased (Exline et al., 2012). Accordingly, we proposed the following hypothesis:

H5: *In the presence of travel companions, tourists from collectivistic cultures are more likely to have a greater range of culinary selections than tourists from individualistic cultures.*

Moreover, the work of Clauzel et al. (2019) reported that the number of guests at a party and the relationships among these guests significantly influence food consumption behavior. Further, in the case of set menus, couples are the greatest consumers while those who take meals alone account for the minimal number of orders. Similarly, Cheng et al. (2013) revealed that in the presence of an opposite-gender companion, a consumer is more likely to purchase impulsively. Accordingly, we proposed:

H6: *In the presence of an* opposite-gender *partner, tourists from collectivistic cultures are more likely to consume a greater range of culinary selections than tourists from individualistic cultures.*

Previous studies identify that the perceived social distance within the group members significantly determines the effects of the social influence on the final consumption decisions (Akerlof, 1997; Cheng et al., 2013). However, the nature of the social influence effect is determined by the values of companions. For example, family members and friends may discourage impulsive consumption by avoiding wastefulness and extravagance, while socially more distant peer shoppers are less likely to come up with comparable concerns for long-term consequences. Thus, socially more distant peers may trigger unplanned purchases that satisfy hedonic goals and spontaneity (Luo, 2005). In the context of dining, social pressure induces more food intake (Exline et al., 2012), for example dining with colleagues and unfamiliar travelers may predict more interpersonal concern compared with friends and family members. Accordingly, we proposed the following hypothesis:

H7: *In the presence of socially more distant travel companions, tourists from collectivistic cultures are more likely to consume a greater range of culinary selections than tourists from individualistic cultures.*

### Data and Methods

The data for Study 2 was based on two secondary sources, one data was from the original work of Hofstede (2015), and the other one was obtained from the Osaka Convention and Tourism Bureau covering for 2 years. Data for the primary independent variables, the cultural dimensions of UAI and IDV were adopted from the original work of Hofstede (2015), the survey respondents were assigned the score based on their place of origin. Among those 112 centuries and regions, the score of UAI ranged from 8 the lowest (Singapore) to 112 the highest (Greece).

Data for other variables were obtained from the Osaka Convention and Tourism Bureau (OCTB) who organized seasonal surveys that were administered to international tourists from over 60 countries, from 2016 to 2018 in the international departure hall of Kansai International Airport. The survey was originally written in Japanese and was then translated to five languages by experts (English, Japanese, Simplified Chinese, Traditional Chinese, and Korean), and conducted during eight periods as shown in Table 4. The survey consists of 37 questions, including social demographics (age, gender, and nationality), travel attributes (packaged tour or not, travel experience, length of stay in Osaka, and travel companions), and travel activities (purchased items, culinary consumption, sightseeing spots, and entertainment activities). The self-administered seasonal questionnaire was administered to 9,141 random samples of tourists from different countries/regions to Osaka, Japan. Moreover, 194 samples were eliminated in the empirical analysis due to missing cuisine content or national information, remaining 8,947 samples. Additionally, OCTB also surveyed international tourists whether stayed and dined in Osaka. The culinary consumption behaviors of international tourists tend to be homogeneous, this is caused by the airport context-based dining is fewer selection options compared with center city. Therefore, samples that without staying in Osaka just departing from Kansai Airport were also eliminated in the analysis and finally remained 5,438 usable samples.

**TABLE 4:** Survey overview (*n* = 9,141).

Start date	Last date	Samples
July 19, 2016	July 30, 2016	1452
October 31, 2016	November 11, 2016	1036
January 19, 2017	January 30, 2017	957
March 10, 2017	March 21, 2017	1029
May 23, 2017	June 3, 2017	1008
August 31, 2017	September 11, 2017	1384
November 30, 2017	December 11, 2017	1236
February 22, 2018	March 5, 2018	1039

To concretely explore the culinary consumption of international tourists, a multiple-choice question with 23 choices encompassing all possible restaurant categories was designed. Those 23 types of restaurants include both Japanese cuisines and non-Japanese cuisines, Japanese cuisines mainly eat separately, while non-Japanese cuisines, such as Chinese cuisines often shared. Considering the type of food that may have influences on food sharing behaviors, to address this critical issue, we conducted the analysis by omitting non-Japanese cuisines to make sure the robustness of the results. Finally, based on the 16 restaurant categories, this study designed three dependent variables (*Japanese daily food*, *Japanese luxury food*, and *the total number of cuisines consumed*). The detailed information of cuisines is presented in the first column of Table 1. If at least one of the cuisines was selected, it was assumed that respondents had consumed the respective food and the value of “1” was assigned for consumed categories, otherwise “0.” Travel companions (*partners*, *family*, *friends*, and *others*) were treated as moderating variable strings. In addition, we define the composite variable to simplify the discussion. Let *companion* = 1 if a respondent travels with either partner, family, friends, and/or colleague (other).

Because packaged tour or not, travel experiences, consumption per day, and length of stay are expected to have different culinary consumption patterns and extent of culinary knowledge, we control for these effects a consumption per day (total spending/nights of stay in Japan); packaged tour or not (tour = 1; individually = 0); first/repeat trip to Osaka (first time = 1; repeat = 0); length of stay in Japan/Osaka (0–7 nights = 0–7, 8–13 nights = 10.5; 14 or more = 14). The control variables are also including the age, gender (woman = 1), satisfaction (very dissatisfied = 1; very satisfied = 7), and revisit intention (strongly disagree = 1; strongly agree = 5) of the respondents.

### Results

**FIGURE 8:**
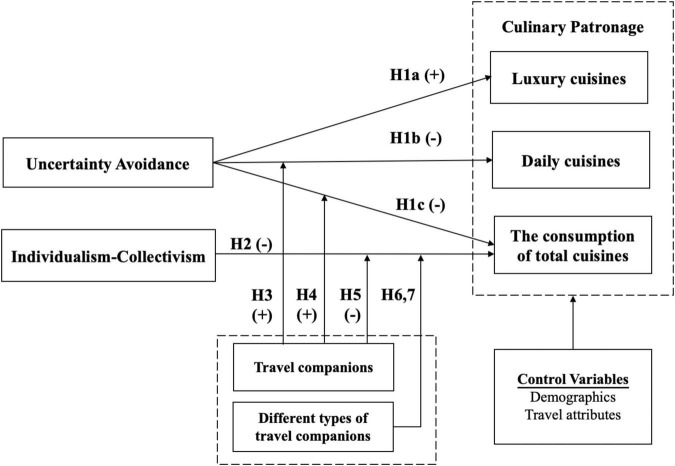
Conceptual framework.

A total of 5,438 usable responses were obtained for data analysis, with 3703 excluded due to missing cuisine content or nationality information, or without staying in Osaka. Table 5 shows the summary statistics of the variables. In the sample, roughly 50% of tourists were from three Asian countries/regions, China (22%), South Korea (17%), and Taiwan (10%) followed by the U.S. (6%). Figure 8 presents the shares of nations/regions of international tourists to Osaka from 2016 to 2018.

**TABLE 5:** Summary statistics and correlation matrix (*n* = 5,438).

	Mean	*SD*	Lux	Daily	Total	UAI	IDV	Comp	Spent	Age	Gen	1st	Pack
Luxury foods	0.786	0.410											
Daily foods	0.938	0.241	0.162*										
Total experiences	4.741	2.622	0.476*	0.379*									
UAI	0.000	1.000	0.000	0.039*	0.046*								
IDV	0.000	1.000	−0.093*	−0.155*	−0.139*	−0.053*							
Companion	0.814	0.389	0.079*	0.125*	0.098*	−0.035*	−0.220*						
Consumption per day (log)	5.378	1.833	–0.019	−0.032*	−0.043*	−0.185*	0.056*	0.003					
Age (log)	3.226	0.394	0.002	−0.150*	−0.093*	−0.076*	0.203*	−0.041*	0.080*				
Gender (female = 1)	0.530	0.499	–0.002	0.067*	–0.016	−0.107*	−0.165*	0.135*	0.036*	−0.068*			
First Trip (first trip = 1)	0.133	0.340	–0.012	−0.057*	−0.095*	−0.158*	−0.063*	0.109*	0.075*	0.044*	0.017		
Package (tour = 1)	0.612	0.487	–0.018	−0.035*	−0.035*	−0.035*	0.045*	0.043*	0.044*	−0.101*	0.029*	0.021	
Stay nights	3.780	2.004	0.181*	0.133*	0.353*	−0.091*	0.000	−0.072*	−0.187*	−0.045*	–0.007	−0.170*	−0.039*

*UAI, Uncertainty avoidance; IDV, Individualism/Collectivism.*

*p < 0.05.

#### Results for the Main Effect

We used the binomial logit model and Poisson regression model to estimate the national/regional culture on culinary preference for luxury cuisines, daily cuisines, and total culinary consumption. Those analyses were conducted in R (version 4.0.5)^1^. The binomial logit model is often used as a vector of random variables to a binomial random variable (Coull and Agresti, 2015). We used the binomial decision data of tourists which was grouped culinary data consisting of counts of successes in luxury cuisines group or daily cuisines group. Accordingly, the binomial logit model was an appropriate method to test H1a, H1b, and H3. Additionally, we applied the Poisson regression model to estimate the influence of national/regional culture on the total culinary consumption of tourists. Poisson regression is often used for modeling count data (Cameron and Trivedi, 1990), and therefore this model is feasible to test H1c H2, H4, and H5, and the results are shown in Table 6. There were some other candidate models, such as the binomial probit model instead of the logit model, and the negative binomial regression instead of the Poisson regression. However, the logit model and Poisson regression model provided robust results and was widely used for binomial dependent variables and non-negative integer dependent variables. We examined the model based on the relationships that our hypothesis should indicate.

**TABLE 6:** Estimation results of the binomial logit and Poisson regression models (*n* = 5,438).

Dependent variables	M1-0 Luxury			M1-1 Luxury			M2-0 Daily			M2-1 Daily			M3-0 Total			M3-1 Total		
						
Model	Logit			Logit			Logit			Logit			Poisson			Poisson		
						
	Est	*SE*		Est	*SE*		Est	*SE*		Est	*SE*		Est	*SE*		Est	*SE*	
(Intercept)	–0.477	0.330		–0.477	0.330		5.818	0.593	***	5.840	0.595	***	1.217	0.062	***	1.205	0.062	***
County culture																		
UAI	0.063	0.035	+	–0.016	0.074		0.196	0.065	**	0.020	0.106		0.040	0.007	***	0.028	0.016	+
IDV													–0.071	0.007	***	–0.048	0.013	***
Travel Companion																		
Companion	0.548	0.084	***	0.541	0.084	***	1.133	0.129	***	1.149	0.130	***	0.173	0.017	***	0.179	0.018	***
Control variables																		
Consumption per day	0.031	0.019	+	0.032	0.019	+	0.025	0.032		0.029	0.032		0.017	0.004	***	0.017	0.004	***
Age	0.092	0.087		0.091	0.087		–1.479	0.154	***	–1.494	0.155	***	–0.074	0.016	***	–0.073	0.016	***
Gender	–0.059	0.069		–0.056	0.069		0.372	0.121	**	0.377	0.122	**	–0.047	0.013	***	–0.046	0.013	***
First trip	0.108	0.102		0.115	0.102		–0.327	0.157	*	–0.301	0.158	+	–0.080	0.020	***	–0.079	0.020	***
Package	–0.067	0.070		–0.067	0.070		–0.439	0.127	***	–0.441	0.127	***	–0.027	0.013	*	–0.027	0.013	*
Stay nights	0.268	0.020	***	0.268	0.020	***	0.315	0.037	***	0.318	0.037	***	0.099	0.003	***	0.099	0.003	***
Interactions																		
UAI*Companion				0.101	0.083					0.275	0.133	*				0.015	0.017	
IDV*Companion																–0.031	0.016	*
AIC	5418.3			5418.8			2207.9			2205.7			24578.4			24577.4		

*UAI, uncertainty avoidance; IDV, individualism/Collectivism; Est, estimates; SE, standard error.*

+p < 0.1, *p < 0.05, **p < 0.01, ***p < 0.001.

First, we examined the overall fitness of the models. According to AIC shown in Table 4, the overall fitness of the without interaction model (M1-0) is better than with interaction model (M1-1) to explain Luxury cuisine consumption, while with interaction models (M2-1, M3-1) are better to explain Daily and Total consumption. Therefore, we examine the result of models M1-0, M2-1, and M3-1. Consistent with hypothesis H1a, the relationship of UAI with the consumption of Japanese luxury cuisines remained positive and significant at a 10% level (M1-0, β = 0.063, *p* < 0.1). However, the hypothetic negative relationship between UAI and consumption of Japanese daily cuisines was not supported (M2-1, β = 0.020, *p* > 0.1). Therefore, Hypothesis 1b is not supported. H1c aims to estimate the influence of UAI negatively affects the range of culinary consumption, whereas the result is in the opposite direction of H1c (M3-1, β = 0.028, *p* < 0.1), and thus H1c is not supported. Moreover, results reveal that tourists from collectivistic cultures are more inclined to consume a broad range of culinary types at the destinations (M3-1, β = –0.048, *p* < 0.001), and the results are consistent with what we hypothesized, thus H2 is supported.

### Results Examining the Effects of Different Types of Companions

Afterward, we applied the Poisson regression model to examine H6 and H7, and the main aim was to examine whether different types of travel companions (partner, family, friend, and others) have varied impacts on the relationship between collectivism and consumption of daily cuisines.

**FIGURE 9:**
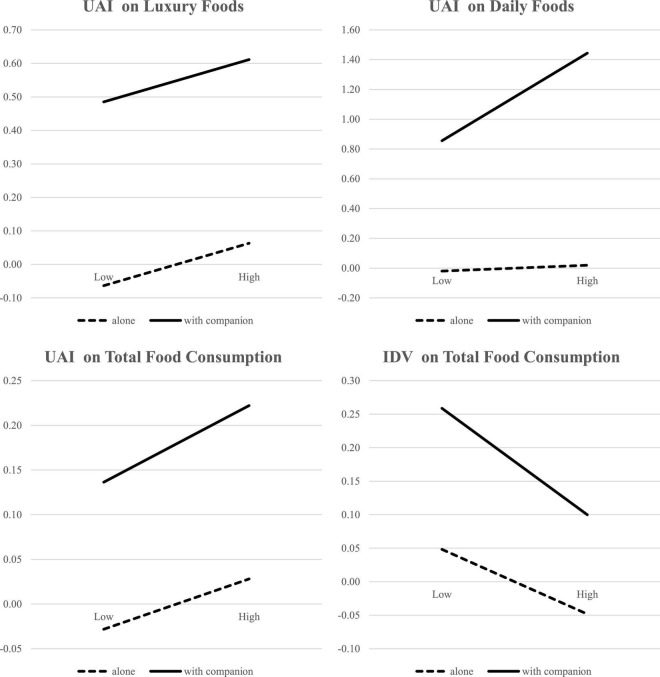
The moderating effect of travel companion. UAI, uncertainty avoidance; IDV, individualism-collectivism; Low IDV, collectivism; High IDV, individualism.

To test the hypotheses H6 and H7, we referred to the results of model M4-1 which was well fitted (lower AIC) for the dependent variables compared with M4-0. H6 was hypothesized that the social presence of a partner significantly moderates the relationship between collectivism and the range of culinary consumption (M4-1, β = –0.07, *p* < 0.001), and thus H6 was supported. Additionally, H7 was hypothesized that the presence of socially more distant travel companions significantly moderates the relationship between collectivism and the range of culinary consumption (M4-1, β = –0.110, *p* < 0.01), and thus H7 was supported. Results for the moderating effects of different types of travel companions are shown in Table 7. While the results of all hypotheses are presented in Table 8 and Figure 9.

**TABLE 7:** Estimation results of the Poisson regression models (*n* = 5,438).

Dependent variables	M4-0 Total cuisines			M4-1 Total cuisines		
		
Models	Poisson			Poisson		
		
	Estimates	*SE*		Estimates	*SE*	
(Intercept)	1.256	0.062	***	1.233	0.063	***
Country culture						
UAI	0.042	0.007	***	0.026	0.014	.
IDV	–0.074	0.007	***	–0.052	0.013	***
Travel companions						
Partner	0.191	0.022	***	0.202	0.023	***
Family	0.156	0.017	***	0.163	0.018	***
Friend	0.133	0.017	***	0.140	0.017	***
Others	0.059	0.036	.	0.052	0.037	
Demographics						
Consumption per day	0.016	0.004	***	0.017	0.004	***
Age	–0.079	0.017	***	–0.076	0.017	***
Gender	–0.048	0.013	***	–0.043	0.013	***
First trip	–0.076	0.020	***	–0.076	0.020	***
Package	–0.026	0.013	*	–0.026	0.013	*
Stay nights	0.098	0.003	***	0.098	0.003	***
Interactions						
UAI × Partner				0.004	0.022	
UAI × Family				0.004	0.017	
UAI × Friend				0.038	0.017	*
UAI × Others				0.017	0.036	
IDV × Partner				–0.070	0.020	***
IDV × Family				–0.013	0.018	
IDV × Friend				–0.005	0.018	
IDV × Others				–0.110	0.040	**
AIC	24572.9			24562.6		

*UAI, uncertainty avoidance; IDV, individualism/collectivism.*

^+^p < 0.1, *p < 0.05, **p < 0.01, ***p < 0.001.

**TABLE 8:** Result of hypotheses estimation.

Hypothetic relationship	Expectations	Results	
H1a: High-UAI to luxury food choice	UAI: Positive	Positive	Supported
H1b: Low-UAI to daily food choice	UAI: Negative	N.S.	Not supported
H1c: Low-UAI to total food choice	UAI: Negative	Positive	Not supported
H2: Collectivism to total food choice	IDV: Negative	Negative	Supported
H3: Companion moderating of high-UAI to daily food choice	UAI: Positive	Positive	Supported
H4: Companion moderating of high-UAI to total food choice	UAI: Positive	N.S.	Not supported
H5: Companion moderating of collectivism and total food choice	IDV: Negative	Negative	Supported
H6: Partner moderating of collectivism and total food choice	IDV: Negative	Negative	Supported
H7: Others moderating of collectivism and total food choice	IDV: Negative	Negative	Supported

## Discussion and Conclusion

Two empirical studies with different types of data and methods show the cross-cultural differences of tourist culinary experiences and behaviors at Osaka, based on UAI and IDV. Study 1 reveals the differences in the topics discussed by the two cultural dimensions. First, the high-UAI group show more interest in discussing topics related to luxury cuisines, whereas the low ones focused on daily local cuisines. Moreover, tourists from the high-UAI group seemed to dine out with travel companions (such as sister, husband) and had explored a greater range of food than the low ones. Second, tourists from individualistic cultures focused on food quality and waiting time, whereas tourists from collectivistic cultures centered on economic aspects, such as price or set food.

Study 2 reveals that tourists from high-UAI cultures tend to consume luxury cuisines, while those from lower ones are more inclined to consume local novel and inexpensive cuisines. Moreover, tourists from collectivistic cultures appear to have a greater range of culinary choices. Specifically, tourists from high high-UAI cultures accompanied by friends induce them to patronage a greater range of local cuisines and “play with safe” of changing inexpensive daily food. Tourists from collectivistic cultures along with the presence of travel companions (partner versus family versus friend versus colleague) show a greater range of food choices than those traveling alone. The travel attributes information further reveals that repeat-visitors showed a stronger patronization of Japanese daily cuisines than did first-time visitors; younger tourists appear greater variety-seeking behavior in unfamiliar cuisines. This research thus provides significant theoretical and practical implications.

### Theoretical Implications

Our research thus offers several important theoretical implications for advancing food tourism literature by revealing the cultural differences in tourist culinary exploration behaviors. Additionally, this study focuses on how social facilitation impacts national culture and the culinary consumption behaviors of tourists to shed light on how interpersonal factors impact the consumption decision-making of tourists.

First, our study uncovered that the national culture of UAI and IDV has an impact on multiple aspects of tourist culinary exploration, including the consumption of luxury versus daily cuisine, and the range of culinary consumption. Tourists from high-UAI cultures are more likely to consume luxury cuisines, given that they have a stronger need for clarity and security, and formal dining environments provide a sense of assurance (De Mooij and Hofstede, 2002). Tourists from low-UAI cultures are more risk-taking, enjoy adventure-seeking activities, and thus they are more likely to try novelty cuisines (Tse and Crotts, 2005). In contrast, tourists from high-UAI cultures tend to be risk-avoiding, and they tend to avoid unfamiliar cuisines, and specifically distant the street food and the food in night markets. Usually, compared with luxury cuisines, daily cuisines may less secure, especially raw dishes and/or cold dishes. Therefore, tourists from high-UAI cultures are more likely to consume Japanese luxury cuisines than daily cuisines. However, tourists from higher-UAI cultures tend to have a greater range of culinary consumption, which indicates that a high level of food security or high-end offering, such as luxury cuisines and/or high food quality tends to induce the culinary consumption of tourists. Our results contrast with the common assumption that tourists from high-UAI cultures have a smaller range of culinary consumption (Tse and Crotts, 2005), which is a Hong Kong-based context, Hong Kong cuisines were profoundly influenced by Chinese cuisines; thus, tourists from different regions (i.e., the United States, mainland China, Taiwan, and Singapore) come with different levels of prior knowledge on Hong Kong food, and the food culture of tourists greatly influences their food perception and evaluation of destination cuisines (Chang et al., 2011).

Second, the study results indicated that individualism/collectivism has a different influence pattern, in that tourists from collectivistic cultures showed a greater range of food consumption than those from individualist cultures. This finding can be explained by their tendency of collectivist preference to travel with family members, friends, and other travelers rather than alone, and can also be explained by their value for social bonds with travel companions. The results are in line with the collectivist value which emphasizes the importance of dining to maintain or improve relationships with others (Hui and Triandis, 1986).

Third, our findings identified that travel companion moderate the relationship between national culture and culinary consumption. Prior studies examined the novelty culinary-seeking behaviors of the international tourists (Tse and Crotts, 2005) or the uncertainty avoidance of immigrants on their attitudes toward local foods (Seo et al., 2012), while the literature on how cultural background affect the culinary consumption behaviors of tourists is relatively neglected. Our findings shed insight on those tourists from low-UAI cultures are more likely to consume a greater range of local daily cuisines (novelty culinary-seeking behaviors) while dining with travel companions. Additionally, in the presence of travel companions, tourists from collectivistic cultures will have a significant effect on the consumption of a wider range of culinary selections than tourists from individualistic cultures. The social facilitation of eating seems to be working in such a situation (Luo, 2005). Dining together helps to create an atmosphere of relaxation and a positive mood, and food becomes more palatable, leading to greater exploration of the local food (Kim and Huang, 2021). There is also a “time extension” effect when people eat together (De Castro et al., 1990), and social interaction reduces self-monitoring, thus tourists may engage more in the exploration of food (Bellisle and Dalix, 2001).

Fourth, our study contributed to the literature in identifying the moderating effects of different types of travel companions on the relationship between national culture and culinary consumption. The moderating effect of opposite partners and less familiar others are both positive and significant, while the two are distinct in terms of social distance. Firstly, the results of this study indicate tourists from collectivistic cultures show a greater range of culinary consumption when accompanied by opposite partners, the finding is in line with Townsend and Levy (1990), their findings heightened preference for opposite partners with resources is expected to be more attractive. Indeed, females perceived men as more desirable when they exhibit signals to resource access (Dunn and Searle, 2010). In the literature on consumer behavior, Cheng et al. (2013) reveal that an opposite-gender companion is more likely to induce impulse consumptions. Clauzel et al. (2019) report that the proportion of set menus ordered by a couple is mostly maximized, while it is minimized for those who take meals alone. Our study further revealed that those tourists from collectivistic cultures dining with socially more distant travel companions (such as colleagues and unfamiliar travelers) would increase the total number of culinary consumptions. Our findings are consistent with the existing literature of Luo (2005), which states that companions with far social distance less tend to come up with comparable concerns for long-term consequences, thus socially more distant peers may trigger impulsive consumption by satisfying hedonic goals and spontaneity. Our findings also connected with the findings of Jaffe et al. (2019) in that social distance positively and significantly influences the consumption of individuals regarding diversity.

Finally, our study further revealed a statistically significant relationship between travel experiences and culinary exploration behaviors, including positive relationships between travel experiences and novelty-seeking and variety-seeking behaviors on culinary exploration. This is consistent with the findings of Mak et al. (2012) that with more overseas travel experiences, tourists generally have a broader exposure to novel and foreign cuisines.

### Managerial Implications

This study used both online and offline large and real international samples to conduct the cross-cultural comparison of the culinary consumption of tourists (e.g., luxury versus daily versus variety-seeking behaviors). The findings provided destination managers with a better understanding of the culinary preferences of international tourists and choice context and develop marketing communication strategies effectively. This study has several practical implications for destination marketing management.

First, the study findings suggested that luxury cuisines together with excellent service and environment should be leveraged to attract repeat tourists from high-UAI cultures. In the case of Japanese cuisines, tourism marketers should promote Kaiseki, Fugu, crab, and similar traditional local luxury cuisines to fascinate tourists from Belgium, Greece, Portugal, and other high-UAI scored travel markets.

Second, this study could provide practical implications for operators to extend travel companions to induce a greater range of culinary consumption. Our findings reveal that those tourists from collectivistic cultures are more likely to consume a greater range of cuisines when accompanied by partners and other companions (such as colleagues and unfamiliar travelers). Accordingly, marketers should promote local cuisines and food-related activities to tourists from collectivistic cultures based on the types of travel companions. For example, the recommendation of local alcohol or special cuisines to the international tourists accompanied by a couple and or colleagues would be desirable. Therefore, travel destinations that are rich in culinary resources should leverage those resources to attract tourists from collectivistic cultures, including China, South Korea, and Russia. Moreover, tourists from high-UAI cultures also increase the likelihood of daily cuisines in the context of dining with travel companions. Accordingly, increasing the trust perception of local cuisines is also expected to attract the patronization of tourists from risk-avoiding cultures.

Finally, repeat visitors showed a stronger patronization of Japanese daily cuisines than did first-time visitors. Accordingly, marketers should promote more Japanese daily foods to the highly repeated travel markets, including Hong Kong, Korea, Singapore, and Taiwan. More specifically, younger visitors have a clear tendency to enjoy diverse and unfamiliar cuisines. Therefore, this study provides valuable insights to policymakers to increase financial outcomes. Promoters could develop food travel packages based on travel companions, travel experiences, and age data.

### Limitations and Future Research Directions

Although the current study provided important contributions to applying food tourism as a means of promoting a destination and attracting tourists, there were still certain limitations that should be considered. First, the sample of Study 1 is limited to the User-Generated Content in English. Despite the Chinese tourists with the largest market shared in Japanese inbound tourists, the count of comments by Chinese tourists was much low compared with their proportions. Thus, further research using Chinese social media would provide greater insights. Second, this research focused solely on Osaka Japan, and, therefore, the conclusions might not be generalized. Future research should extend this model to more host countries and regions where food tourism is one of the leading tourism industries to determine the generalizability of the cross-national results. Third, this study was limited to identifying multinational cultures on the culinary consumption of inbound tourists from the macro level, and future research should explore the intrinsic psychological factors of individuals through the additional methodology. Fourth, the cultural distance might significantly impact the culinary knowledge and language familiarity of tourists, and, in turn, impact their culinary consumption, the survey did not cover the information on the host country language skills of respondents and their prior food knowledge of the various culinary offerings. Therefore, future research may include the language skills of the host country and prior food knowledge as controls in the research model. Finally, the design of the survey methodology also needed to be improved. We would be able to obtain more appropriate results by using the share of food consumption, rather than the binary question of “ate” or “did not eat” for luxury food and daily food, as in this study.

## Data Availability Statement

The datasets presented in this article are not readily available because the data is under a non-disclosure agreement. Requests to access the datasets should be directed to the corresponding authors.

## Ethics Statement

Ethical review and approval was not required for the study in accordance with local legislation and institutional requirements. Written informed consent was not required in accordance with local legislation and institutional requirements.

## Author Contributions

BL and YW contributed to overllall quality and supervision the empirical work. SK and XL contributed to the second part of empirical work (i.e., the regression work), to the analysis of the results, and to the writing of the first draft. YL contributed to the data collection and the implementation of the second part of empirical work. WG contributed to bridging two main parts of the empirical analyzes and polishing the manuscript. All authors discussed the results and commented on the manuscript.

## Conflict of Interest

The authors declare that the research was conducted in the absence of any commercial or financial relationships that could be construed as a potential conflict of interest.

## Publisher’s Note

All claims expressed in this article are solely those of the authors and do not necessarily represent those of their affiliated organizations, or those of the publisher, the editors and the reviewers. Any product that may be evaluated in this article, or claim that may be made by its manufacturer, is not guaranteed or endorsed by the publisher.

## Footnotes

1
https://cran.r-project.org/web/packages/
