# Corrigendum: National Culture and Culinary Exploration: Japan Evidence of Heterogenous Moderating Roles of Social Facilitation

**DOI:** 10.3389/fpsyg.2021.824855

**Published:** 2021-12-22

**Authors:** Bin Liu, Yang Wang, Sotaro Katsumata, Yulei Li, Wei Gao, Xi Li

**Affiliations:** ^1^Economics and Management School, Jiangxi Science and Technology Normal University, Nanchang, China; ^2^Greater Bay Area International Institute for Innovation, Shenzhen University, Shenzhen, China; ^3^Graduate School of Economics, Osaka University, Toyonaka, Japan; ^4^Business School, Durham University, Durham, United Kingdom; ^5^Japanese Department, School of Foreign Languages, Shenzhen University, Shenzhen, China; ^6^College of Management, Shenzhen University, Shenzhen, China

**Keywords:** travel companions, user-generated content, culinary consumption, uncertainty avoidance, individualism-collectivism

In the published article, there was an error in the Funding statement. The correct funding information is below.

“This project was supported by the JSPS KAKENHI Grant No. JP21H00757, National Natural Science Foundation of China (Grant Nos. 72102151, 71973044, and 71661009), and Jiangxi Provincial Education Science (Grant No. 21ZD063).”

In the published article, there was an error in affiliation 3. Instead of “School of Economics, Osaka University, Suita, Japan,” it should be “Graduate School of Economics, Osaka University, Toyonaka, Japan.”

Additionally, there has been a change in authorship. Bin Liu and Yang Wang should share first authorship.

In the original article, there was an error in the Ethics statement. The correct Ethics statement is below.

“Ethical review and approval was not required for the study in accordance with local legislation and institutional requirements. Written informed consent was not required in accordance with local legislation and institutional requirements.”

In the original article, there was an error in the Data Availability Statement. The correct Data Availability Statement is below.

“The datasets presented in this article are not readily available because the data is under a non-disclosure agreement. Requests to access the datasets should be directed to the corresponding authors.”

The authors apologize for these errors and state that they do not change the scientific conclusions of the article in any way. The original article has been updated.

## Publisher's Note

All claims expressed in this article are solely those of the authors and do not necessarily represent those of their affiliated organizations, or those of the publisher, the editors and the reviewers. Any product that may be evaluated in this article, or claim that may be made by its manufacturer, is not guaranteed or endorsed by the publisher.

